# Nasal Natural Killer/T-cell Lymphoma with Skin, Eye, and Peroneal Nerve Involvement

**DOI:** 10.5505/tjh.2012.03360

**Published:** 2012-12-05

**Authors:** Burcu Türker, Burak Uz, Metin Işık, Özlen Bektaş, Haluk Demiroğlu, Nilgün Sayınalp, Aysegül Üner, Osman İlhami Özcebe

**Affiliations:** 1 Hacettepe University, Faculty of Medicine, Department of Internal Medicine, Ankara, Turkey; 2 Hacettepe University, Faculty of Medicine, Department of Hematology, Ankara, Turkey; 3 Hacettepe University, Faculty of Medicine, Department of Rheumatology, Ankara, Turkey; 4 Hacettepe University, Faculty of Medicine, Department of Pathology, Ankara, Turkey

**Keywords:** Nasal-type NK/T-cell lymphoma, Radiotherapy, Peroneal nerve, Drop foot

## Abstract

Nasal-type natural killer (NK)/T-cell lymphoma (NKTL) is a rare disease strongly associated with Epstein-Barr virus and is often localized to the upper aerodigestive tract at presentation. Extranodal NKTL may involve any extranodal site and disease beyond the nasal cavity is highly aggressive, with short survival time and poor response to therapy. Herein we present a 57-year-old male that had been treated with systemic chemotherapy and cranial radiotherapy for nasaltype NKTL in the palate with skin, right eye, and right peroneal nerve involvement. He was given salvage chemotherapy consisting of 3 cycles of ICE and his response to the therapy was satisfactory, except for persistent right drop foot. About 6 weeks later, the patient presented with bilateral total loss of vision and proptosis; therefore, DHAP chemotherapy was started. Unfortunately, after 1 cycle of the second salvage chemotherapy, he died due to severe fungal infection of the hard palate.

Despite the fact that involvement of any extranodal site is possible, concurrent involvement of many systems in NKTL patients is unusual. Nasal-type NKTL has a poor prognosis, despite local radiotherapy and systemic chemotherapy. Physicians should be aware of this rare disorder than can only be diagnosed after extensive immunohistochemical studies.

**Conflict of interest:**None declared.

## Abstract (tr)

Nazal tip NK/T-hücreli lenfoma, Epstein-Barr virüs ile yüksek oranda ilişkili ve başvuru anında sıklıkla üst solunum/ sindirim sistemine yerleşen nadir bir hastalıktır. Nazal bölge dışındaki tutulumlar sıklıkla agresif seyirli olup, tedaviye yanıtları kötüdür ve yaşam süreleri oldukça kısadır. Damakta nazal tip NK/T-hücre lenfoma tanısı olup sistemik kemoterapi ve kranial radyoterapi tedavileri uygulanmış bir erkek hasta, cilt, sağ göz ve sağ peroneal sinir tutulumları ile karşımıza çıktı. 3 kür ICE kurtarma kemoterapisi ile başarılı bir yanıt elde edildi, fakat sağda düşük ayak deformitesi devam etti. Yaklaşık 6 hafta sonra her iki gözde tam görme kaybı ve proptozis ile başvurdu. Bunun üzerine DHAP kemoterapisine geçildi. 1 kür sonrası sert damakta meydana gelen fungal enfeksiyon neticesinde hasta ex oldu. Bu tür lenfomalarda herhangi bir bölgenin tutulumu olabilmekle birlikte, aynı hastada birçok bölgenin etkilenmesi sık görülen bir durum değildir. Nazal tip NK/T-hücreli lenfomalarda lokal radyoterapi ve sistemik kemoterapiye rağmen prognoz kötüdür. Klinisyenler ileri immünhistokimyasal çalışmalarla tanı konabilen bu nadir hastalığa karşı uyanık olmalıdırlar.

## INTRODUCTION

Nasal-type NK/T-cell lymphoma (NKTL) is a rare entity strongly associated with Epstein-Barr virus (EBV). The neoplastic cells derived from NK cells and/or cytotoxic T-lymphocytes involve the nasal cavity and paranasal sinuses. NKTL is most prevalent in Asians, and the Native American population of Mexico, Central America, and South America. Irrespective of ethnic origin, EBV is the most probable pathogenetic agent [1]. Extranodal NKTL almost always exhibits extranodal presentation. The disease is often localized to the upper aerodigestive tract at presentation and bone marrow involvement is uncommon. Patients with nasal involvement present with symptoms of nasal obstruction and epistaxis. Lymphoma may extend to adjacent tissues and/or may disseminate rapidly to various sites (e.g. skin, gastrointestinal tract, testis, and cervical lymph nodes). Extranodal NKTL occurring beyond the nasal cavity is highly aggressive, and is associated with a short survival time and poor response to therapy [1]. 

Although any extranodal site involvement may be seen in this disease, concurrent involvement of many systems is unusual. Herein, we present the clinical, morphological, and immunohistochemical features, and clinical findings in a case of relapsed nasal-type NKTL with skin, eye, and peroneal nerve involvement. It is noteworthy that colon adenocarcinoma and NKTL occured in the presented patient, which is unusual and probably had a negative effect on prognosis.

## CASE

A 57-year-old male presented to our outpatient unit in March 2009 with abdominal distention, abdominal pain, and constipation of 15-d duration. To evaluate the onset of constipation colonoscopic evaluation was performed and a tumoral lesion on the ascending colon was noted. Pathological evaluation was compatible with adenocarcinoma, which was extending to the serosa, and right hemicolectomy was performed. Further treatment was not planned, because the surgical margins were clear of tumoral tissue and there was no lymph node metastasis. Additionally, the tumor was moderately differentiated. 

**Figure 1:**
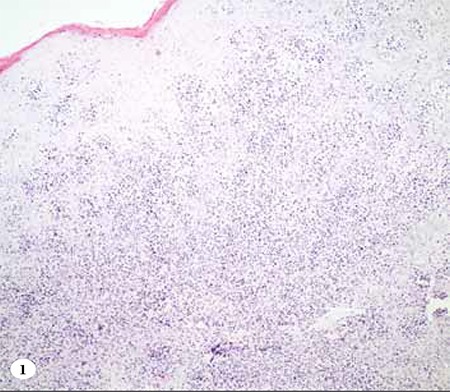
H&E staining of the lesion located on the palates hows abundant lymphocytes.

**Figure 2:**
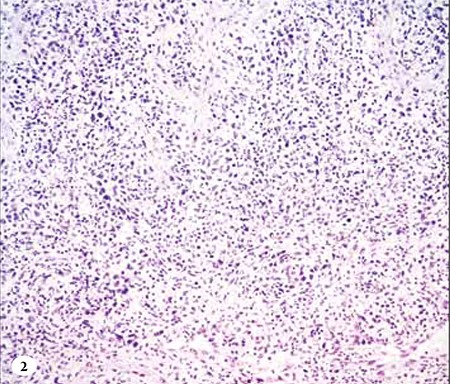
H&E staining of the lesion located on the palate shows abundant lymphocytes.

**Figure 3:**
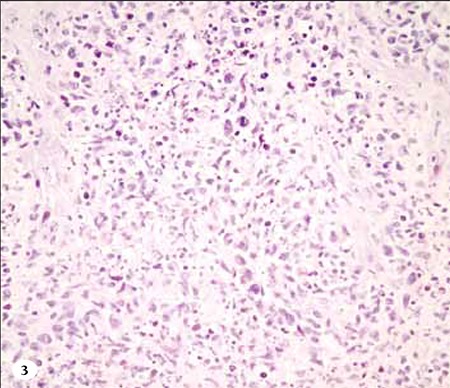
H&E staining of the lesion located on the palate shows abundant lymphocytes.

Four months later the patient was re-evaluated due to a new lesion on the palate. The white, painful lesion was rapidly enlarging and had a tendency to bleed. In a short time, a hard immobile 3-cm mass on the right cheek appeared and the patient began to complain of nasal congestion, dyspnea, and fever, which were especially severe at night. He also lost 5 kg of body weight, and was diagnosed as sinusitis at a different hospital and was treated with antibiotics. Because there was no clinical improvement, incisional biopsy was performed and nasal-type NKTL was diagnosed (Figures 1,2,3); the initial stage was III ESB, according to the Ann-Arbor staging system. International Prognostic Index (IPI) score at presentation was 0 (low risk). Serum ELISA tests were positive for EBV EBNA IgG (47.5 RU mL^–1^) (normal range: 0-20 RU mL–^1^) and EBV VCA IgG (147.7 RU mL^–1^) (normal range: 0-20 RU mL^–1^). 

**Figure 4:**
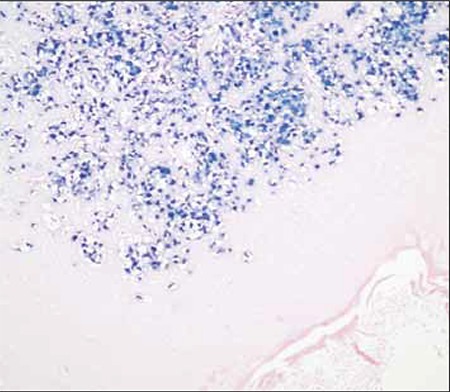
The presence of EBV small RNAs in the neoplastic cells was observed via in situ hybridization using EBV-encoded small RNA (EBER) oligonucleotides.

The presence of EBV small ribonucleic acids (RNAs) in the neoplastic cells was observed via in situ hybridization using EBV-encoded small RNA (EBER) oligonucleotides (Figure 4). Neoplastic cells stained positive for CD3, CD56, and granzyme B, and negative for CD20. The Ki-67 proliferation index was 80%-90%. Nasopharyngeal MRI showed a giant lesion measuring 6.1x4.9x7.1cm located in the nasal cavity and the palate that extended to the pterygopalatine fossa, filling the nasopharynx and the oropharynx, and narrowing the airway. Furthermore, bilateral cervical lymphadenopathies, which increased in size and in metastatic nature, were observed. Abdominal CT showed a splenic hypodense mass measuring 17 mm, but thoracic CT and bone marrow biopsy were normal. 

Colon biopsy specimens that were obtained at the time adenocarcinoma was diagnosed were re-evaluated and determined to be negative for lymphoma involvement. As such, chemotherapy and local radiotherapy were scheduled. On 1 September 2009 we initiated chemotherapy (CHOP regimen: cyclophosphamide, vincristine, adriamycin, and prednisolone) in 21-d cycles plus central nervous system (CNS) prophylaxis with methotrexate 15 mg t.w. for 3 weeks. Following 3 cycles of CHOP, 3 cycles of cranial radiotherapy were administered. Cervical and thoracic CT post treatment were normal. Follow-up abdominal CT showed that the splenic mass had decreased in size (1 cm). Colonoscopic evaluation was performed again and the findings were normal. 

**Figure 5:**
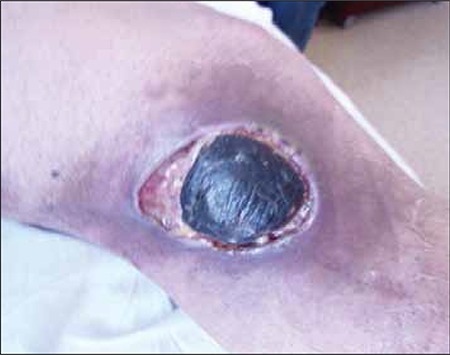
Lesion on the right knee.

**Figure 6:**
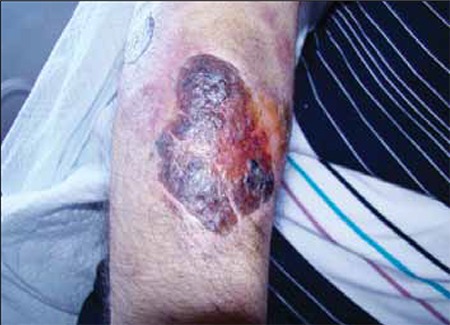
Lesion on the right arm.

**Figure 7:**
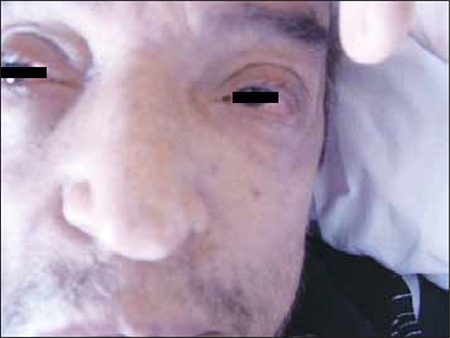
The patient’s eyes, hyperemia, and edema.

**Figure 8:**
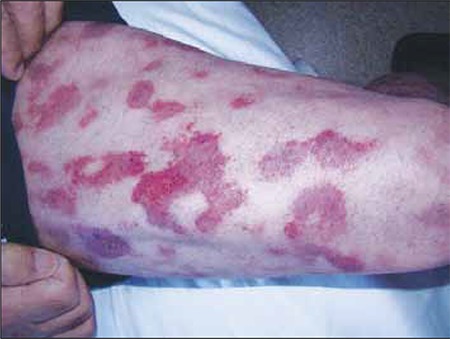
Paraneoplastic lesions on the legs that ocurred during the course of chemotherapy.

On 11 June 2010 the patient developed erythematous lesions on his arms and legs, which increased in diameter and ulcerated during the days that followed (Figures 5 and 6). E. Coli and S. Haemolyticus were noted in culture specimens and IV antibiotic therapy was started. A skin biopsy specimen was obtained and reported as leukemic infiltration. Immunohistochemical examination of neoplastic cells showed diffuse positivity with CD56 staining. On 21 June 2010 the patient developed right drop foot. MRI of the right knee showed increased contrast involvement in the peroneal nerve trace, indicative of a malignant infiltrate. Electromyographic findings were compatible with an axonal lesion in the proximal region of the peroneal nerve. On 6 July 2010 vision in his right eye deteriorated; orbital MRI showed subretinal hemorrhage, edema, and thickening of the eyelids, which were more prominent in the right eye. He was thought to have exudative retinal detachment as a result of tumor infiltration (Figure 7). The 4x4 cm lesion in his right knee was debrided and improved with therapy. He was then given 3 cycles of ICE (iphosfamide, carboplatin, and etoposide) chemotherapy and 6 cycles of intrathecal chemotherapy. During the first cycle of chemotherapy skin lesions regressed within a few days, but new erythematosus lesions appeared, resembling a drug eruption. Although biopsy specimens from these lesions were not obtained, they were thought to be paraneoplastic and regressed during the course of chemotherapy (Figure 8). In addition, conjunctival hyperemia and corneal epithelial defects regressed. 

**Figure 9:**
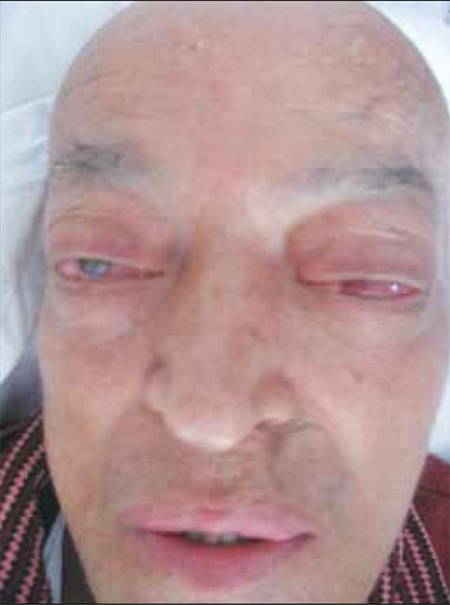
Photograph taken a few days before the patient died shows bilateral proptosis and conjunctival chemosis.

**Figure 10:**
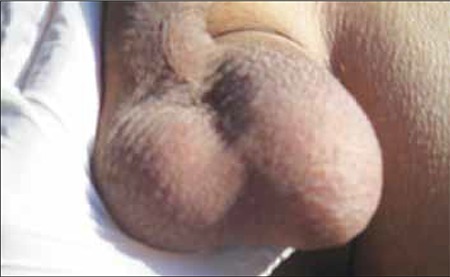
Swelling of the left testis.

Unfortunately, the patient’s general condition deteriorated rapidly during a 2-week period and he developed bilateral total loss of vision, conjunctival chemosis, and proptosis (Figure 9). In addition, he had swelling on his left testis (Figure 10). Scrotal ultrasound showed bilateral thickening of the tunica layers and a right-sided varicocele. Orbital MRI showed a mass lesion surrounding the bilateral bulbus oculi that extended throughout the optic nerves to the retrobulbar region, infiltrating the medial and superior rectus muscles. This lesion showed significant progression, as compared to previous MRI findings. Conjunctival biopsy showed atypical lymphoid cells with significant nucleoli. Some of these cells stained positive for CD3 and CD56, and a small number of scattered cells stained positive for granzyme B. Salvage chemotherapy with DHAP (dexamethasone, cytarabine, and cisplatin) was then initiated. After 1 cycle of chemotherapy a crusty necrotic lesion appeared on the hard palate. A biopsy specimen taken from this lesion showed fungal infection, probably associated with rhinocerebral mucormycosis; therefore, posaconazole treatment was scheduled, but the patient died within a few days.

## DISCUSSION

NKTL is a predominantly extranodal malignancy that is most commonly positive for EBV and CD56, and is usually located in the nasal cavity or paranasal sinuses [1]. Nasaltype NKTL is an aggressive disorder with a tendency to invade local tissues and metastasize to the CNS. The presented patient had nasal congestion, dyspnea, and fever, and subsequently developed chronic nasal obstruction and bleeding. These non-specific symptoms may cause a delay in diagnosis, as in the presented patient. Diagnosis of the presented patient was based on a combination of morphology, positive granzyme B and CD56 expression, and negative staining for B-cell markers (such as CD20 and CD79a). The presence of EBV small RNAs was observed via in situ hybridization using EBER oligonucleotides; however, the cells expressed both CD56 and granzyme B. An unusual feature of the presented case was the expression of CD3, which might have been due to the presence of CD3 molecule zeta chain cytoplasmically in NK cells, that would not be detected on the cell surface by flow-cytometry, but can occasionally reach the detection threshold for immunohistochemical analysis, which can detect surface and cytoplasmic expression simultaneously. 

Rodriquez et al. reported the involvement of unusual extranodal sites, such as the testes, lungs, liver, CNS, bone marrow, and peripheral blood in 6 of 10 patients that presented with local disease [2]. Apart from the nasal cavity, the skin is the most common site of involvement, and may be a primary or secondary manifestation of the disease; nearly 10%-20% of patients with nasal lymphomas also have skin involvement [1]. Our patient presented to hospital with skin, eye, and peroneal nerve involvement 3 months after the last cycle of CHOP chemotherapy. Skin biopsy findings were compatible with leukemic involvement; therefore, salvage chemotherapy with ICE was initiated a second time. The patient’s skin lesions improved rapidly in response to this therapy. 

The close proximity of the nasal cavity, paranasal sinuses, and eyes may by why the presented patient had ocular metastasis. Primary intraocular T-cell and NKTL are extremely rare and primarily represent as a secondary manifestation of either a cutaneous or systemic lymphoma [3]. They accounted for <3% of all primary ocular lymphoproliferative lesions in reported series [4,5,6]. Hon et al. reported that 6 of 24 primary nasal and nasopharyngeal NKTL patients suffered vision-threatening complications [7]; therefore, examination of the eyes is very important in the diagnosis and management of this type of lymphomatous disease. 

Neurologic deficits are rare in patients with NKTL. Nasal-type NKTL presents with transverse myelopathy and third cranial nerve palsy [8,9]. More recently, nasaltype NKTL of the cauda equina has been reported [10]. In the presented patient right-sided peroneal nerve involvement with drop foot was diagnosed on the basis of clinical, MRI, and electromyographic findings. The pathogenesis of this involvement might have been due to ischemia secondary to angiocentric and angiodestructive infiltration by lymphoma cells [1]. 

The prognosis of nasal NKTL is variable. Unfavorable prognostic factors include advanced-stage disease (stage III or IV), unfavorable IPI, bone or skin invasion, an elevated circulating EBV DNA level, and the presence of EBVpositive cells in bone marrow [1]. The presented patient had a severe clinical course, despite having a low-risk IPI. Although patients with low IPI scores (≤1) have better survival rates, the prognostic significance of the IPI score is not as consistent in NKTL patients as in those with other types of aggressive NHL [11]. A new prognostic model for NKTL defined by Na et al. was constructed using the LDH level, performance status, B symptom, and stage [12]. This patient was allocated to group 4 according to his weightage scores, and his 1-year survival and median survival time were 0% and 3 months, respectively. Measurement of the circulating viral DNA load in peripheral blood is useful for diagnosis, monitoring, and prognostication of the disease [13]. EBV-DNA can therefore be used as a marker to predict tumor burden [14,15], but prediction can potentially be affected by the presence of EBV unrelated to lymphoma [13]. 

Extranodal NKTL occurring beyond the nasal cavity is highly aggressive and is associated with short survival time and poor response to therapy. Historically, the survival rate is poor (30%-40%), but survival has improved in recent years due to the use of more intensive therapy, including upfront radiotherapy. Local radiotherapy is the mainstay of treatment, whereas additional multi-agent chemotherapy improves local disease control and survival [16]. Despite intensive radiotherapy, local failure accounts for 50% of NKTL relapses [17]. 

Nasal NKTL located in the palate is a rare entity, which is diagnosed based on extensive immunohistochemical studies. Nonetheless, it should be kept in mind that if a lesion on the palate or in the nasal cavity is resistant to treatment, malignancy should be an alternative diagnosis. In addition, baseline and regular ophthalmic, neurologic, and testicular assessment is warranted for this type of lymphoma, particularly in patients with NKTL. Unfortunately, nasal-type NKTL has a poor prognosis, despite local radiotherapy and systemic chemotherapy. 

**Conflict of Interest Statement**

None of the authors has any conflicts of interest, including specific financial interests, relationships, and/ or affiliations, relevant to the subject matter or materials included in this manuscript. Written informed consent was obtained from the patients’ wife.
